# First-in-Human Evaluation of Positron Emission Tomography/Computed Tomography With [^18^F]FB(ePEG12)12-Exendin-4: A Phase 1 Clinical Study Targeting GLP-1 Receptor Expression Cells in Pancreas

**DOI:** 10.3389/fendo.2021.717101

**Published:** 2021-08-19

**Authors:** Hiroyuki Fujimoto, Naotaka Fujita, Keita Hamamatsu, Takaaki Murakami, Yuji Nakamoto, Tsuneo Saga, Takayoshi Ishimori, Yoichi Shimizu, Hiroyuki Watanabe, Kohei Sano, Norio Harada, Hiroshi Nakamura, Kentaro Toyoda, Hiroyuki Kimura, Shunsaku Nakagawa, Mitsuharu Hirai, Atsushi Murakami, Masahiro Ono, Kaori Togashi, Hideo Saji, Nobuya Inagaki

**Affiliations:** ^1^Radioisotope Research Center, Agency of Health, Safety and Environment, Kyoto University, Kyoto, Japan; ^2^Department of Diabetes, Endocrinology and Nutrition, Graduate School of Medicine, Kyoto University, Kyoto, Japan; ^3^Department of Diagnostic Imaging and Nuclear Medicine, Graduate School of Medicine, Kyoto University, Kyoto, Japan; ^4^Department of Patho-Functional Bioanalysis, Graduate School of Pharmaceutical Sciences, Kyoto University, Kyoto, Japan; ^5^Research and Development Division, Arkray, Inc., Kyoto, Japan; ^6^Department of Clinical Pharmacology and Therapeutics, Kyoto University Hospital, Kyoto, Japan

**Keywords:** β-cell imaging, exendin-4, glucagon-like peptide-1 receptor (GLP-1R), PET, first-in-human study

## Abstract

Pancreatic β-cell mass (BCM) has a central importance in the pathophysiology of diabetes mellitus. Recently, pancreatic β-cell-specific imaging, especially positron emission tomography (PET) with exendin-based probes, has emerged for non-invasive evaluation of BCM. We developed a novel exendin-based probe labeled with fluorine-18, [^18^F]FB(ePEG12)12-exendin-4 (^18^F-Ex4) for PET imaging. We subsequently conducted a first-in-human phase 1 study of ^18^F-Ex4 PET/computed tomography (CT) and investigated the safety and utility for visualizing the pancreas. Six healthy male subjects were enrolled in this study. A low dose (37.0 MBq) of ^18^F-Ex4 PET/CT was administered (first cohort: n = 2), and subsequently a higher dose (74.0 MBq) was administered (second cohort: n = 4). In the first and second cohorts, 38.6 ± 4.8 and 71.1 ± 4.8 MBq of ^18^F-Ex4 were administered, respectively. No serious adverse events were observed in both groups. Only one participant in the first cohort showed transient hypoglycemia during the PET scans. ^18^F-Ex4 PET/CT successfully visualized the pancreas in all participants. The mean standardized uptake value of the pancreas was found to be higher than that in the surrounding organs, except for the bladder and kidney, during the observation. Dosimetry analyses revealed the effective systemic doses of ^18^F-Ex4 as 0.0164 ± 0.0019 mSv/MBq (first cohort) and 0.0173 ± 0.0020 mSv/MBq (second cohort). ^18^F-Ex4 PET/CT demonstrated the safety and utility for non-invasive visualization of the pancreas in healthy male subjects. ^18^F-Ex4 is promising for clinical PET imaging targeting pancreatic β cells.

## Introduction

The number of diabetic patients is on the rise all over the world. The estimated prevalence of diabetic patients, no less than 20 years old, has risen from 151 million to 463 million between 2000 and 2019. The number is predicted to increase to 578 million and 700 million by 2030 and 2045, respectively (1). In order to control its increase, it is necessary to have a detailed understanding of the pathogenesis of diabetes and to develop new diagnostic strategies and policies for the treatment of the disease.

The pathogenesis of type 2 diabetes mellitus involves failure of insulin secretion and insulin resistance. Although evaluation of the pancreatic β cell function is mandatory to understand the failure of insulin secretion, the systemic β-cell function consists on not only the ability of insulin secretion of individual β cells but also pancreatic β-cell mass (BCM). Actually, BCM has central importance in the progression of diabetes and responsiveness of anti-diabetic treatment (2). Some autopsy and histological studies reported reduced BCM in patients with impaired fasting blood glucose (FBG) levels and/or with type 2 diabetes mellitus (3–5). However, these conventional histological methods of BCM analysis require invasiveness and are limited to cross-sectional observations of partial pancreatic tissues (6). Therefore, there is a need to develop non-invasive methods to determine changes in BCM of the whole pancreas over time in patients with diabetes mellitus. In order to solve this issue, pancreatic β-cell-specific imaging using nuclear medicine techniques has been developed in recent years (7–11). In particular, radioisotope-labeled probes using exendin peptides—including glucagon-like peptide-1 receptor (GLP-1R) agonist and antagonist—have been promising for positron emission tomography (PET) and single-photon emission computed tomography (SPECT) imaging (12–14), which enabled mouse pancreas imaging, its image analysis for BCM quantification (15, 16), and investigation of the protective effect of pancreatic β cells on anti-diabetic treatment (17, 18). In addition, these probes were shown to be useful for the detection of insulinoma (13).

This study reports the results of the first-in-human PET study with [^18^F]FB(ePEG12)12-exendin-4 (^18^F-Ex4) (19), a novel exendin probe labeled with fluorine-18 (^18^F). The purpose of this study is to demonstrate the clinical safety, whole-body distribution, and dose estimation of the probe in healthy subjects, as well as to show the clinical utility for human pancreas imaging by PET.

## Materials and Methods

### Subjects

Inclusion criteria for this study were healthy male subjects (20 years or older) without apparent impaired glucose tolerance (FBG ≤ 100 mg/dl and HbA1c ≤ 5.5%). Exclusion criteria were as follows: body mass index (BMI) ≤18.5 or ≥30.0 kg/m^2^, severe renal failure [estimated glomerular filtration rate (eGFR) ≤30 ml/min/1.73 m^2^], apparent existence of pancreatic tumor or pancreatitis based on evaluation—including ultrasonography and serological findings such as serum amylase levels—or past history of any pancreatic diseases including pancreatitis or pancreatic surgery. This study was approved by the Ethics Committee of Kyoto University Graduate School, Kyoto University Faculty of Medicine, and Kyoto University Hospital (approval no. C1269) and was registered at the University Hospital Medical Information Network Clinical Trials Registry as UMIN000025288. The study was conducted in accordance with the ethical standards of the Declaration of Helsinki (as revised in Fortaleza, Brazil, October 2013). All subjects provided written informed consent for participation in the study.

### Synthesis of ^18^F-Ex4 Probe

**Figure 1:**
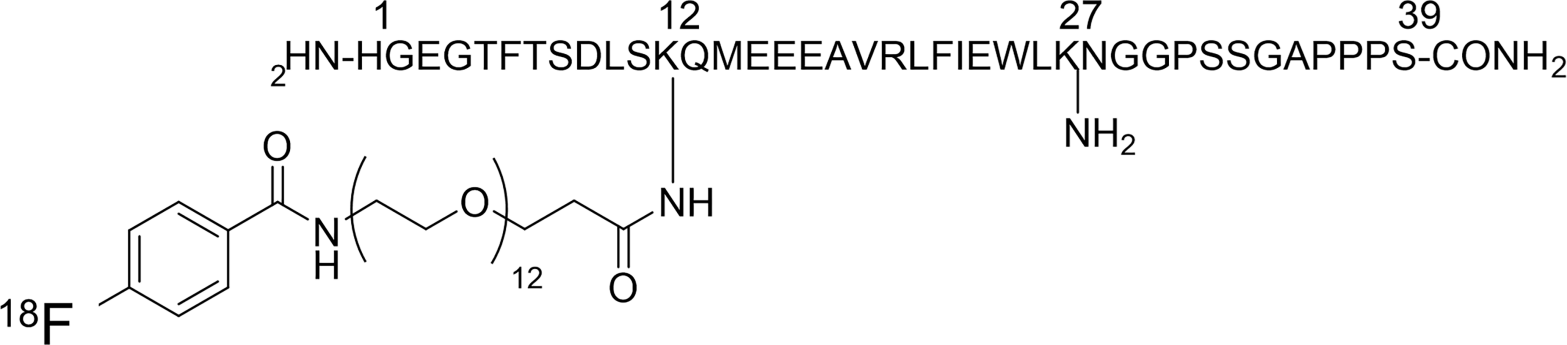
A chemical structure of [^18^F]FB(ePEG12)12-exendin-4.

We synthesized ^18^F-Ex4 probe using an in-house automated synthesis equipment. **Figure 1** shows the structure of ^18^F-Ex4. Probe synthesis consisted of two steps: 1) synthesis of succinimidyl[^18^F]-4-fluorobenzoate ([^18^F]SFB) and 2) condensation reaction of [^18^F]SFB and exendin-4.

[^18^F]Fluoride was produced by a cyclotron (CYPRIS HM-12; Sumitomo Heavy Industries Ltd., Tokyo, Japan) using ^18^O(p,n)^18^F nuclear reaction with proton irradiation of an enriched [^18^O]H_2_O target and passed through an anion exchange solid phase cartridge (Sep-Pak Accell Plus QMA Plus Light Cartridge, Waters Co., Milford, MA, USA). [^18^F]Fluoride was eluted with a potassium carbonate (1.7 mg, 12 μmol) and Kryptofix 222 (7 mg, 18.7 μmol) (Merck, Darmstadt, Germany) in acetonitrile (580 μl) (Nacalai Tesque, Kyoto, Japan). The solvent was removed at 120°C under argon gas flow, the residue of which was azeotropically dried with acetonitrile (880 μl) at 120°C under argon gas flow. Subsequently, 4-(ethoxycarbonyl)-*N*,*N*,*N*-trimethylbenzene ammonium triflate (4.4 mg, 11.4 μmol) (NARD, Kobe, Japan) in acetonitrile (500 μl) was added and then heated at 90°C for 10 min. Tetrapropylammonium hydroxide (Tokyo Chemical Industry, Tokyo, Japan) solution (10% in water, 50 μl) was added and heated at 90°C for 5 min. The solution was cooled after adding *N*,*N*,*N*′,*N*′-tetramethyl-*O*-(*N*-succinimidyl)uranium tetrafluoroborate (15 mg, 50 μmol) (Tokyo Chemical Industry, Tokyo, Japan) in acetonitrile (200 μl). The solution was diluted with 10 mM of acetic acid-sodium acetate buffer (Fujifilm Wako Pure Chemical Corporation, Osaka, Japan). The synthesized [^18^F]SFB was separated by reversed-phase high-performance liquid chromatography (RP-HPLC) using a COSMOSIL PBr column (10 × 250 mm, 5 μm) (Nacalai Tesque, Kyoto, Japan), followed by loading onto an activated Sep-Pak C18 (Sep-Pak C18 environmental cartridge; Waters Co., Milford, MA, USA) and SCX (Bond Elut Jr-SCX; Agilent, CA, USA) cartridges. The [^18^F]SFB was then eluted with acetonitrile (2 ml).

A solution of peptide precursors, pre(ePEG12)12-Ex4 (0.50 mg) (KNC Laboratories Co. Ltd., Kobe, Japan) in acetonitrile/buffer containing 0.5 M of dipotassium hydrogenphosphate (Fujifilm Wako Pure Chemical Corporation) was added to the reaction vessel of [^18^F]SFB. The reaction mixture was incubated at 60°C for 15 min. After 4-methylpiperidine (20 μl) (Fujifilm Wako Pure Chemical Corporation) was added, the solution was further incubated at 60°C for 10 min. The synthesized ^18^F-Ex4 was purified by RP-HPLC using a YMC Triart C8 column (10 × 250 mm, 5 μm) (YMC, Kyoto, Japan) and loaded onto an activated Sep-Pak C18 (Sep-Pak light C18 cartridge; Waters Co., Milford, MA, USA). Finally, ^18^F-Ex4 was eluted with ethanol (Fujifilm Wako Pure Chemical Corporation). The eluent was diluted by saline containing 0.1% polysorbate.

### ^18^F-Ex4 PET/CT Study

The PET/computed tomography (CT) studies were conducted using an integrated PET/CT scanner (Discovery IQ; GE Healthcare, Waukesha, WI, USA) with a bismuth germinate scintillator arranged in five rings and a 16-detector-row CT scanner. The vital signs (blood pressure, pulse rate, and body temperature) and blood glucose level (GT-1670; Arkray, Kyoto, Japan) were checked in the subjects. Before administration of the probe, a low-dose CT was conducted for attenuation correction and image fusion without oral/intravenous contrast. The filter-sterilized ^18^F-Ex4 was slowly administered intravenously for 5 min. The first whole-body PET scan was started immediately after ^18^F-Ex4 administration, and following five-time whole-body scans consisting of 2 min/frame × 6 frames (PET 1–6) were undertaken. The final whole-body scan of 3 min/frame × 6 frames was started at 2 h after administration of ^18^F-Ex4 (PET 7). The vital signs were evaluated at 1 and 2 h after ^18^F-Ex4 administration, at the final PET scan, and 24 h after ^18^F-Ex4 administration. The blood glucose levels were measured at 1, 5, 10, 30, 60, and 120 min after ^18^F-Ex4 administration, as well as at the end of the scanning. Serious hypoglycemia was defined as blood glucose level below 54 mg/dl (20).

A probe of 37.0-MBq dose was to be administered on the first two subjects (first cohort) to ascertain that the probe would not cause serious adverse events. Subsequently, a probe of twice the dosage (74.0-MBq dose) was to be administered on the other four subjects (second cohort).

### Radioactivity in Blood and Urine

Blood collection was performed at 0, 1, 5, 10, 30, 60, and 120 min after ^18^F-Ex4 administration. Urine collection was performed at 0, 60, and 120 min after ^18^F-Ex4 administration. Blood samples collected from subjects were divided into whole blood and plasma samples. The weight and radioactivity count using γ-counter (2480 Wizard Perkin Elmer, Waltham, MA, USA) were measured in both samples. The radioactivity and volume of the urine samples were measured. All count data were decay-corrected to the start time of the probe administration.

### PET/CT Image Analysis and Radiation Dosimetry

Images of the whole body from head to thigh were reconstructed using an ordered subset expectation maximization algorithm (subsets, 14; iteration, 4) with CT-attenuation correction. Point spread function correction was also performed. The field of view in the axial direction was 600 mm, the matrix size was 192 × 192, and the voxel size was 3.1 × 3.1 × 3.3 mm. As for quantification of organ accumulation of ^18^F-Ex4, volumes of interest (VOIs) were generated on various normal organs by referring to the corresponding CT images, and the mean standardized uptake value (SUVmean) was obtained on a workstation (Advantage Workstation 4.6, GE Healthcare).

The software OLINDA/EXM version 1.1 implemented with the Medical Internal Radiation Dose (MIRD) S value was used for radiation dosimetry with data derived from imaging studies. The SUVmean in each organ at each measurement time was converted to percentages of the injected dose per ml (%ID/ml). %ID/ml was multiplied by the volume of each organ to generate percentages of the injected dose per organ at each time point. Time–activity curves of each source organ were then generated, and time-integrated activity coefficients were obtained. From these values, the organ absorbed doses and effective doses were calculated.

### Statistical Analysis

Continuous variables are reported as means ± standard deviations unless otherwise stated. JMP version 13.0.0 (SAS Institute, Cary, NC, USA) was used to perform all analyses.

## Results

### Subjects

Six healthy male subjects (mean age, 22.0 ± 0.9 years) who met all the protocol-specified inclusion criteria and none of the exclusion criteria participated in this study. **Table 1** shows the baseline characteristics of the participants. In the first cohort, 38.6 ± 4.8 (Case 1, 33.9; Case 2, 43.4) MBq of the probe was administered to two test subjects to confirm that the probe would not cause serious adverse events. In the second cohort, 71.1 ± 4.8 (Case 3, 70.7; Case 4, 71.8; Case 5, 64.2; Case 6, 77.6) MBq of the probe was administered to the other four test subjects.

**Table 1:** The baseline characteristics of the participants.

Case no.	Case 1	Case 2	Case 3	Case 4	Case 5	Case 6
Cohort	1st cohort		2nd cohort			
Probe dose (MBq)	33.9	43.4	70.7	71.8	64.2	77.6
Age (years)	21	21	22	22	23	23
BMI (kg/m^2^)	20.1	18.7	20.0	20.8	21.7	19.7
FPG (mg/dl)	84	80	68	67	81	77
HbA1c (%)	5.4	5.1	5.2	5.1	5.3	5.0
eGFR (ml/min/1.73 m^2^)	94.3	88.7	93.0	91.9	113.0	99.3
Amylase (U/L)	74	67	78	58	103	53

BMI, body mass index; FPG, fasting plasma glucose; eGFR, estimated glomerular filtration rate.

### Clinical Safety Evaluation

Overall, the probe was administered safely in all subjects without any serious adverse events. Blood glucose levels tended to fall until 30 min after administration but spontaneously returned to normal without administration of glucose (blood glucose levels after ^18^F-Ex4 administration were 0 min = 88.7 ± 5.3 mg/dl, 1 min = 72.0 ± 12.7 mg/dl, 5 min = 80.3 ± 10.1 mg/dl, 10 min = 73.2 ± 7.7 mg/dl, 30 min = 63.8 ± 8.6 mg/dl, 60 min = 77.3 ± 5.8 mg/dl, 120 min = 77.3 ± 3.4 mg/dl, and at the end of the scans = 77.8 ± 3.4 mg/dl). Only in Case 2 of the first cohort, whose blood glucose level before ^18^F-Ex4 administration was 80 mg/dl, was hypoglycemia (46 mg/dl) without clinical symptoms observed 30 min after probe administration. Serious hypoglycemia was not observed in any of the four subjects in the second cohort. There were no changes in vital signs before, during, or after the PET/CT scans in all six subjects.

### Whole-Body Distribution and Dose Estimation

**Figure 2:**
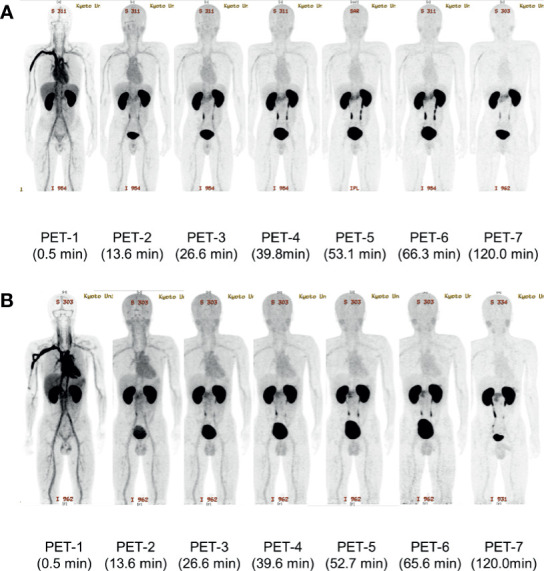
The representative maximum intensity projection images of [^18^F]FB(ePEG12)12-exendin-4 (^18^F-Ex4) PET/CT. The maximum intensity projection images of each PET scan in Case 2 **(A)** and Case 3 **(B)** are shown. The first PET scan was performed immediately after ^18^F-Ex4 administration and following five-time scans consisting of 2 min/frame × 5 frames (PET 1–6) were undertaken. The final scan was started at 2 h after administration of ^18^F-Ex4 (PET 7). The numbers in parentheses indicate the time after ^18^F-Ex4 administration.

**Figure 3:**
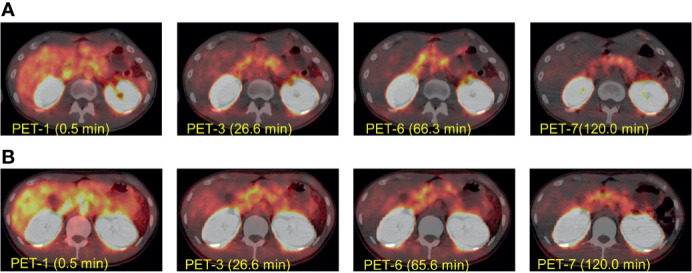
The representative transverse [^18^F]FB(ePEG12)12-exendin-4 (^18^F-Ex4) PET/CT images. The transverse [^18^F]FB(ePEG12)12-exendin-4 (^18^F-Ex4) PET/CT images of Case 2 **(A)** and Case 3 **(B)**. The first PET scan was performed immediately after ^18^F-Ex4 administration, and following five-time scans consisting of 2 min/frame × 5 frames (PET 1–6) were undertaken. The final scan was started at 2 h after administration of ^18^F-Ex4 (PET 7). The numbers in parentheses indicate the time after ^18^F-Ex4 administration.

^18^F-Ex4 PET/CT successfully visualized the pancreas in all participants. Whole-body maximum intensity projection images of PET 1–7 in Cases 2 and 3 are illustrated in **Figure 2** as representative images of the first and second cohorts, respectively. **Figure 3** shows transverse PET–CT fusion images of Cases 2 and 3 at the level of the pancreas. The probe showed strong accumulation in the bladder and kidneys, which are its excretory pathways, after administration. The pancreas, the target organ of this probe, was adequately depicted throughout the time of observation. In addition, the time-dependent decrease in the probe accumulation in the surrounding organs led to a clearer delineation of the pancreas with time. Although this tendency was similar in both cohorts at different doses, a clearer picture of the pancreas was obtained 120 min later in the first cohort compared with the second cohort.

**Figure 4:**
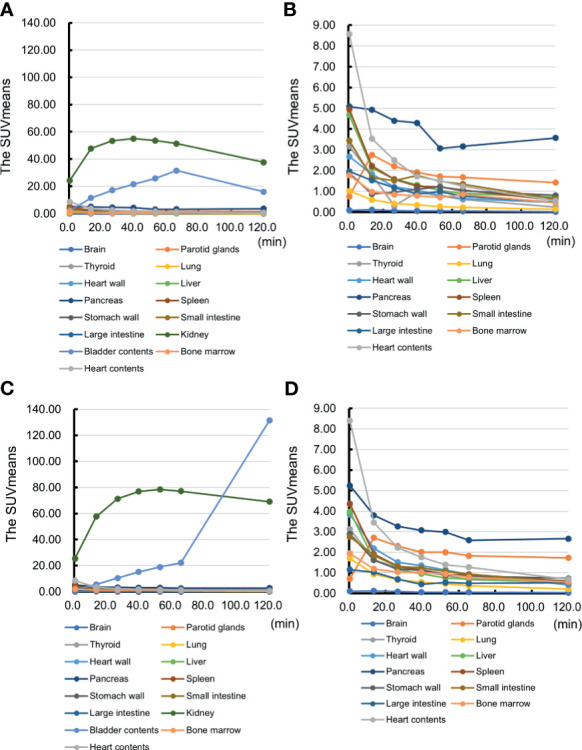
The representative time course of the mean standardized uptake value (SUVmean) in each organ. The time course of the mean standardized uptake value (SUVmean) in each organ of Case 2 and Case 3 is shown. Case 2: **(A)** the SUVmeans of all the organs and **(B)** the SUVmeans excluding kidney and bladder contents. Case 3: **(C)** the SUVmeans of all the organs and **(D)** the SUVmeans excluding kidney and bladder contents.

The SUVmeans of the pancreas also showed the reasonably high values during the scans in all participants. **Figure 4** shows the time course of the SUVmeans in each organ in representative cases of the first and second cohorts. The values of the pancreas were higher than those in the surrounding organs, except for the kidneys, during the time of observation. In addition, the pancreas-to-organ uptake ratios also demonstrated sustainably high values during the observation (**Table 2**). These values can underlie the well-depicted pancreas and the pattern changes on PET/CT images.

**Table 2:** The pancreas-to-organ uptake ratios of [^18^F]FB(ePEG12)12-exendin-4 (^18^F-Ex4) in the first and second cohorts.

		Pancreas/liver		Pancreas/spleen		Pancreas/kidneys		Pancreas/stomach		Pancreas/small intestine		Pancreas/large intestine	
		Mean	SD	Mean	SD	Mean	SD	Mean	SD	Mean	SD	Mean	SD
**1st cohort**	PET 1	1.48	0.55	1.34	0.45	0.25	0.06	4.97	3.22	3.24	2.50	2.68	0.13
	PET 2	2.16	0.22	2.31	0.13	0.09	0.01	3.89	2.42	3.18	0.29	3.27	0.07
	PET 3	3.14	0.52	3.21	0.60	0.08	0.01	3.54	1.47	4.81	2.71	3.70	0.00
	PET 4	3.68	0.57	3.51	0.11	0.08	0.00	4.57	1.00	2.94	0.73	5.56	0.96
	PET 5	4.18	1.74	3.19	0.91	0.07	0.01	3.69	1.68	3.92	2.62	4.68	2.23
	PET 6	4.31	1.11	3.31	0.49	0.07	0.01	4.00	1.29	3.26	1.29	6.07	2.76
	PET 7	5.04	0.11	4.99	1.65	0.08	0.01	4.38	0.05	4.77	1.15	6.83	0.86
**2nd cohort**	PET 1	1.25	0.10	1.23	0.05	0.22	0.03	2.47	1.11	2.30	0.48	3.52	0.76
	PET 2	2.02	0.36	2.20	0.29	0.08	0.02	2.83	1.68	2.27	0.51	2.99	0.54
	PET 3	2.76	0.18	3.17	0.61	0.07	0.02	3.24	1.62	2.74	0.34	4.39	2.24
	PET 4	2.98	0.30	4.09	1.15	0.06	0.02	3.06	1.74	2.84	0.32	4.93	1.75
	PET 5	3.52	0.64	4.40	0.96	0.06	0.02	2.90	1.22	3.21	0.62	4.06	1.46
	PET 6	3.71	0.34	4.76	1.41	0.06	0.02	3.21	1.74	3.57	0.60	3.81	1.88
	PET 7	4.72	0.56	5.96	1.68	0.06	0.02	4.06	0.74	4.98	0.36	5.21	2.32

**Table 3** shows the results of dosimetry (mSv/MBq) in all participants in the first and second cohorts. The highest absorbed doses were found in the excretory pathways: the kidney (first cohort, 0.2420 ± 0.0028 mSv/MBq; second cohort, 0.2955 ± 0.0072 mSv/MBq) and the bladder (first cohort, 0.0867 ± 0.0032 mSv/MBq; second cohort, 0.0765 ± 0.0021 mSv/MBq). The pancreas demonstrated reasonable accumulations (first cohort, 0.0245 ± 0.0026 mSv/MBq; second cohort, 0.0229 ± 0.0020 mSv/MBq), whereas the surrounding organs showed substantially low absorbed doses. The effective doses were 0.0164 ± 0.0019 mSv/MBq and 0.0173 ± 0.0020 mSv/MBq for the first and second cohorts, respectively.

**Table 3:** The results of dosimetry of [^18^F]FB(ePEG12)12-exendin-4 (^18^F-Ex4) in the first and second cohorts.

Dosimetry(mSv/MBq)	1st cohort		2nd cohort	
Target organ	Mean (×10^−1^)	SD(×10^−2^)	Mean (×10^−1^)	SD(×10^−2^)
Adrenals	0.116	0.021	0.130	0.185
Brain	0.010	0.014	0.010	0.014
Breasts	0.028	0.042	0.029	0.035
Gallbladder wall	0.089	0.003	0.095	0.081
Small intestine	0.082	0.080	0.086	0.105
Stomach wall	0.123	0.191	0.101	0.040
Upper large intestine wall	0.085	0.001	0.084	0.071
Lower large intestine wall	0.072	0.009	0.070	0.026
Heart wall	0.113	0.106	0.126	0.071
Kidneys	2.420	0.283	2.955	7.190
Liver	0.117	0.191	0.115	0.059
Lungs	0.043	0.030	0.048	0.027
Muscle	0.046	0.028	0.046	0.021
Pancreas	0.245	0.255	0.229	0.195
Red marrow	0.063	0.016	0.068	0.058
Osteogenic cells	0.060	0.056	0.062	0.055
Skin	0.029	0.036	0.030	0.028
Spleen	0.145	0.120	0.149	0.250
Testes	0.043	0.001	0.041	0.050
Thymus	0.036	0.049	0.037	0.042
Thyroid	0.047	0.020	0.061	0.063
Urinary bladder wall	0.867	3.161	0.765	2.075
Total body	0.058	0.017	0.061	0.027
Effective dose	0.164	0.191	0.173	0.196

## Discussion

This study is a first-in-human study of PET/CT targeting GLP-1R expression cells in the pancreas using a novel ^18^F-Ex4 probe. The main objectives of this study were clinical safety assessment and probe utility for non-invasive pancreatic imaging in humans. None of the six healthy male subjects who participated in the present study experienced any serious adverse events. Only one participant demonstrated transient hypoglycemia without any symptoms, and he recovered spontaneously. Moreover, ^18^F-Ex4 PET/CT successfully visualized the pancreas in all cases.

As the early clinical trials of nuclear medicine probes targeting the pancreas, PET imaging with ^18^F-fluoropropyl-dihydrotetrabenazine was investigated (8), but there remains a concern that accumulation of the probes in peripancreatic organs may interfere with the accurate assessment of the pancreatic β cells. Therefore, the possibility of pancreatic imaging using exendin has received a lot of attention and has been explored by several groups including us (10, 13–15). So far, several groups have reported clinical studies using exendin-based probes, including ^111^In-labeled ones for SPECT (21, 22) and more recently ^68^Ga-labeled ones for PET imaging (23, 24). However, in order to extend our knowledge on the role of BCM in the pathophysiology of type 1 and 2 diabetes, more effective and safer probes are required, especially for repeated examinations in cases with benign diseases like diabetes mellitus and endogenous hyperinsulinemia (24). We therefore developed ^18^F-Ex4 for PET imaging since ^18^F is one of the most widely used radioisotopes in today’s clinical settings and is suitable for cost-efficient high-volume synthesis and delivery.

This study achieved successful visualization of the pancreas *via* maximum intensity projection (**Figure 2**) and PET/CT images (**Figure 3**). The uptakes of the probe in the surrounding organs did not affect visualization and analysis of the pancreas. Taken together with time-dependent analyses of SUVmeans (**Figure 4**) and the pancreas-to-organ uptake ratios (**Table 2**), the probe accumulations decreased sufficiently in the organs around the pancreas until 2 h after administration. Therefore, it seems possible that adjusting the PET scanning time can prevent effects from the surrounding organs.

Two different doses of ^18^F-Ex4 were investigated in this study. Severe adverse events did not increase even at a higher dose of ^18^F-Ex4 (71.1 ± 4.8 MBq in the second cohort), while hypoglycemia was observed only in the lower dose cohort (38.6 ± 4.8 MBq in the first cohort). The hypoglycemia was mild, with no subjective symptoms. Based on the clearer visualization and higher probe accumulation of the pancreas in the first cohort (**Figures 3** and **Table 2**), 37 MBq of ^18^F-Ex4 was sufficient for clinical pancreatic imaging.

It is also necessary to show the safety of the probe as a clinical diagnostic agent. Calculating radiation doses from the probe is essential to assess its safety for clinical use. The effective systemic doses of ^18^F-Ex4 were 0.0164 ± 0.0019 mSv/MBq (0.61 ± 0.07 mSv/37 MBq) (first cohort) and 0.0173 ± 0.0020 mSv/MBq (1.28 ± 0.01 mSv/74 MBq) (second cohort) (**Table 3**), which were comparable with ^18^F-2-fluoro-2-deoxy-d-glucose (^18^F-FDG) (0.019 mSv/MBq, 3.52 mSv/185 MBq) (25), and apparently lower than other exendin-based probes (22). Therefore, ^18^F-Ex4 probe can be used safely and will not pose any particular health hazard with regard to exposure.

Probe accumulation in the kidney was greater than that in the pancreas; accumulation in the kidney is therefore relevant to visualizing the pancreas. **Figure 3** shows that probe accumulation in the kidney is decreased in a time-dependent manner, which did not affect visualization of the pancreas. However, we need to investigate this issue in additional normal, healthy subjects.

The purpose of this study was to evaluate the safety of the probe and to depict the pancreas; prediction of the number of pancreatic β cells has not yet been studied. We focus on the expression of GLP-1R because our probe accumulates in organs *via* GLP-1R. In the pancreas, GLP-1R is expressed not only in pancreatic β cells but also in exocrine cells (26), etc. We plan further study of using SUV for predicting the number of pancreatic β cells taking into consideration accumulating cases.

Finally, this is a phase 1 study with a limited number of healthy male subjects. Further clinical studies in patients affected by specific diseases, such as type 1 and 2 diabetes, insulinoma, and/or congenital hyperinsulinemia, are warranted to establish the clinical safety and utility of the probe. Any such study should be conducted with blood glucose monitoring during the procedure and sufficient attention to the hypoglycemic symptoms.

In conclusion, this phase 1 study of ^18^F-Ex4 PET/CT demonstrates the reasonable safety and utility of ^18^F-Ex4 for non-invasive visualization of the pancreas in healthy male subjects. ^18^F-Ex4 is a promising probe for clinical PET imaging and can have the potential to be a useful tool for the evaluation of BCM in both healthy subjects and patients with diabetes, if found to be specific for β cells.

## Data Availability Statement

The original contributions presented in the study are included in the article/supplementary material. Further inquiries can be directed to the corresponding author.

## Ethics Statement

The studies involving human participants were reviewed and approved by Kyoto University Graduate School and Faculty of Medicine, Ethics Committee. The patients/participants provided their written informed consent to participate in this study. Written informed consent was obtained from the individuals for the publication of any potentially identifiable images or data included in this article.

## Author Contributions

HF planned the study, contributed to data analysis and discussions, and drafted and reviewed the manuscript. NF planned the study, collected the data, and contributed to discussions. KH planned the study and collected and analyzed the data. TM planned the study; collected, analyzed, and discussed the data; and wrote, reviewed, and edited the manuscript. YN, TS, and TI collected and analyzed the data and contributed to discussions. YS, HW, and KS contributed to the study plan. NH contributed to the discussion and reviewed the manuscript. HN contributed to the study plan and data acquisition. KeT contributed to the conception of the study and planned the study. HK contributed to the conception of the study. SN contributed to the data acquisition. MH and AM contributed to the conception of the study. MO and KaT contributed to discussions and reviewed the manuscript. HS contributed to discussions and reviewed the manuscript. NI contributed to the study conception and discussions and reviewed and edited the manuscript. All authors contributed to the article and approved the submitted version.

## Funding

This work was supported by the Advanced Research for Medical Products Mining Program of the National Institute of Biomedical Innovation (NIBIO) of Japan; Translational Research Network Program from the Japan Agency for Medical Research and Development (AMED), a Research Grant from the Ministry of Health, Labor, and Welfare of Japan; the Center of Innovation Program from Ministry of Education, Culture, Sports, Science and Technology (MEXT) and Japan Science and Technology Agency (JST); and Grants-in-Aid for Scientific Research (JP 18KO8475) from the Japan Society for the Promotion of Science.

## Conflict of Interest

NI received clinical commissioned/joint research grants from Mitsubishi Tanabe, AstraZeneca, Astellas, and Novartis Pharma and scholarship grants from Takeda, MSD, Ono, Sanofi, Japan Tobacco Inc., Mitsubishi Tanabe, Novartis, Boehringer Ingelheim, Kyowa Kirin, Astellas, Daiichi-Sankyo, and Taisho-Toyama Pharma. TS is the endowed chair of the sponsored-research program by Nihon Medi-Physics Co., Ltd. MH and AM are employed by Arkray, Inc.; and HN was employed by Arkray, Inc.

The remaining authors declare that the research was conducted in the absence of any commercial or financial relationships that could be construed as a potential conflict of interest.

## Publisher’s Note

All claims expressed in this article are solely those of the authors and do not necessarily represent those of their affiliated organizations, or those of the publisher, the editors and the reviewers. Any product that may be evaluated in this article, or claim that may be made by its manufacturer, is not guaranteed or endorsed by the publisher.
